# Hepatic arterial infusion chemotherapy plus tyrosine kinase inhibitors with or without PD-1 inhibitors for advanced hepatocellular carcinoma with VP4 portal vein tumor thrombosis: a retrospective cohort study

**DOI:** 10.3389/fimmu.2026.1832313

**Published:** 2026-05-20

**Authors:** Weifu Liu, Kongzhi Zhang, Shiguang Chen, Xiaolong Wang, Wenchang Yu

**Affiliations:** 1 Department of Oncology and Vascular Interventional Therapy, Clinical Oncology School of Fujian Medical University, Fujian Cancer Hospital, Fuzhou, China; 2 Fujian Provincial Key Laboratory of Tumor Biotherapy, Fuzhou, China

**Keywords:** hepatic arterial infusion chemotherapy, hepatocellular carcinoma, PD-1 inhibitors, portal vein tumor thrombosis, tyrosine kinase inhibitors

## Abstract

**Background:**

Hepatocellular carcinoma (HCC) with VP4 portal vein tumor thrombosis (PVTT) has a poor prognosis. Although hepatic arterial infusion chemotherapy (HAIC) combined with tyrosine kinase inhibitors (TKIs) and programmed cell death protein 1 (PD-1) inhibitors shows activity in advanced HCC with PVTT, whether adding PD-1 inhibitors to HAIC plus TKIs improves outcomes specifically in VP4 PVTT remains unclear.

**Methods:**

This single-center retrospective study enrolled consecutive treatment-naïve patients with advanced HCC and VP4 PVTT who received HAIC plus TKIs (dual therapy) or HAIC plus TKIs and PD-1 inhibitors (triple therapy) from January 2018 to July 2025. Stabilized inverse probability of treatment weighting (sIPTW) was used to minimize selection bias. Primary endpoints were objective response rate (ORR), progression-free survival (PFS), overall survival (OS), and safety. Tumor response was assessed according to Response Evaluation Criteria in Solid Tumors (RECIST) version 1.1.

**Results:**

Ninety-seven patients were included: 43 in the dual therapy group and 54 in the triple therapy group. Median follow-up was 42.3 months. After sIPTW adjustment, triple therapy achieved a higher ORR (58.9% vs 31.4%, P = 0.012) and disease control rate (94.8% vs 72.1%, P = 0.003). PVTT ORR was also higher with triple therapy (49.9% vs 26.5%, P = 0.034). Triple therapy was associated with longer sIPTW-adjusted median PFS (7.3 vs 5.5 months; HR 0.48, 95% CI 0.31–0.75, P = 0.001) and OS (14.6 vs 10.1 months; HR 0.47, 95% CI 0.29–0.74, P = 0.001). Multivariable Cox regression identified treatment regimen and baseline neutrophil-to-lymphocyte ratio as independent prognostic factors for both PFS and OS. Grade 3–4 treatment-related adverse events were comparable (35.2% vs 30.2%, P = 0.606), with no treatment-related deaths.

**Conclusion:**

In patients with advanced HCC and VP4 PVTT, the addition of PD-1 inhibitors to HAIC plus TKIs was associated with improved tumor response and prolonged survival without an apparent increase in severe treatment-related adverse events. These findings support triple therapy as a potentially preferred first-line strategy for this high-risk population. Prospective randomized trials are needed to validate these findings.

## 1 Introduction

Hepatocellular carcinoma (HCC) is one of the most common malignancies worldwide and the third leading cause of cancer-related death (1, 2). Portal vein tumor thrombosis (PVTT) occurs in 44%–62% of HCC patients and is an independent predictor of poor prognosis (3). VP4 PVTT, defined by the Japan Society of Hepatology (JSH) as tumor thrombus involving the main portal trunk or contralateral first-order branch (4), has the worst prognosis among all PVTT subtypes. Without effective treatment, median overall survival (OS) in these patients is only 2.7–4.0 months (5).

Current guidelines recommend immune checkpoint inhibitor (ICI)-based combinations such as atezolizumab plus bevacizumab or tyrosine kinase inhibitors (TKIs) including sorafenib and lenvatinib as first-line therapy for advanced HCC with PVTT (6, 7). However, outcomes remain unsatisfactory in patients with VP4 PVTT. In the IMbrave150 trial, atezolizumab plus bevacizumab achieved a median OS of 7.6 months in patients with main portal vein invasion (8). Although newer ICI-based regimens, including durvalumab plus tremelimumab [HIMALAYA (9)], nivolumab plus ipilimumab [CheckMate 9DW (10)], and camrelizumab plus rivoceranib [CARES-310 (11)], improved survival compared with TKI monotherapy, patients with VP4 PVTT were often excluded or not separately reported, limiting the applicability of these findings to this high-risk population.

Hepatic arterial infusion chemotherapy (HAIC) delivers cytotoxic agents directly via tumor-feeding arteries, achieving high intratumoral drug concentrations while minimizing systemic exposure (12). Phase III trials in patients with high intrahepatic tumor burden demonstrated superior survival with HAIC compared with transarterial chemoembolization (TACE) and sorafenib monotherapy (13, 14), leading to its inclusion in Asian clinical guidelines (4, 15). Subsequent trials showed that HAIC plus sorafenib outperformed sorafenib alone in HCC patients with PVTT (16, 17). More recently, retrospective studies and phase II trials suggest that triple therapy combining HAIC, TKIs, and programmed cell death protein 1 (PD-1) inhibitors may yield median OS exceeding 17 months in PVTT cohorts (18–20).

However, existing evidence on HAIC combined with systemic therapy has important limitations. Most studies included heterogeneous PVTT subtypes, and even those specifically enrolling patients with VP4 PVTT generally compared HAIC-based combination regimens only against HAIC monotherapy, TKI monotherapy, or TKI plus PD-1 inhibitor combinations. Whether adding a PD-1 inhibitor to HAIC plus TKIs provides additional survival benefit in VP4 PVTT remains unclear. We conducted a retrospective study in treatment-naïve patients with advanced HCC and VP4 PVTT, directly comparing HAIC plus TKIs with or without PD-1 inhibitors, to determine whether PD-1 inhibitors improve outcomes in this high-risk population.

## 2 Materials and methods

### 2.1 Study design and patient selection

This single-center retrospective study was approved by the Ethics Committee of Fujian Cancer Hospital (K2025-380-01) and conducted in accordance with the Declaration of Helsinki and institutional ethical standards. All patients provided written informed consent.

We reviewed consecutive treatment-naïve patients with advanced HCC and VP4 PVTT who received HAIC combined with TKIs, with or without PD-1 inhibitors, at Fujian Cancer Hospital from January 2018 to July 2025.

Inclusion criteria were: (a) age 18–75 years; (b) HCC diagnosed according to American Association for the Study of Liver Diseases (AASLD) criteria and/or histopathology (21); (c) radiologically confirmed VP4 PVTT according to the 2021 JSH guidelines (4); (d) no prior HCC treatment; (e) Eastern Cooperative Oncology Group performance status (ECOG PS) 0–2; (f) Child-Pugh class A or B liver function; (g) at least one measurable lesion according to Response Evaluation Criteria in Solid Tumors (RECIST) version 1.1; (h) adequate hematologic and organ function (white blood cell count ≥3.0 × 10^9^/L, neutrophil count ≥1.5 × 10^9^/L, platelet count ≥75 × 10^9^/L, hemoglobin ≥85 g/L; alanine aminotransferase [ALT] and aspartate aminotransferase [AST] ≤5 × upper limit of normal [ULN]; serum creatinine ≤1.5 × ULN); and (i) completion of at least two HAIC cycles.

Exclusion criteria were: (a) contraindications to study treatments such as active infection or severe cardiopulmonary dysfunction; (b) upper gastrointestinal bleeding within 6 months before enrollment; or (c) inadequate imaging for response assessment or incomplete follow-up data.

### 2.2 HAIC treatment

HAIC procedures were performed by experienced interventional radiologists according to consensus recommendations (15, 22). After percutaneous femoral artery catheterization using the Seldinger technique, selective angiography of the celiac trunk and superior mesenteric artery was performed. Potential collateral feeders were assessed, with additional angiography of other vessels (phrenic, right renal, or internal thoracic arteries) as needed. A microcatheter was then positioned in the dominant tumor-feeding artery. In cases with dual arterial supply or accessory feeders, non-dominant branches were embolized with lipiodol or gelatin sponge particles to optimize flow distribution and minimize off-target infusion. The catheter was then connected to an infusion pump for drug delivery.

The HAIC regimen consisted of oxaliplatin (85 mg/m²) infused over 4 hours and raltitrexed (3 mg/m²) over 1 hour, repeated every 3 weeks for up to 6 cycles. HAIC was discontinued for radiographic progression, intolerable toxicity, death, patient withdrawal, or tumor downstaging sufficient for curative resection or ablation. For grade 3–4 toxicities, treatment was held until recovery to grade ≤1, with subsequent cycles administered at a 25% dose reduction.

### 2.3 TKIs treatment

Oral lenvatinib (8 mg daily for body weight <60 kg; 12 mg daily for ≥60 kg) or apatinib (500 mg daily without PD-1 inhibitors; 250 mg daily with PD-1 inhibitors) was started within 3 days after the first HAIC session. TKIs were given continuously, with treatment cycles defined as 3-week intervals aligned with the HAIC schedule. Dose reduction or temporary interruption was permitted for TKI-related toxicities according to standard practice. TKIs were permanently stopped for radiographic progression, unacceptable toxicity, death, or patient withdrawal.

### 2.4 PD-1 inhibitor treatment

PD-1 inhibitors were initiated within 3 days after the first HAIC session. Agents included pembrolizumab, camrelizumab, tislelizumab, and sintilimab, each given intravenously at 200 mg every 3 weeks to align with HAIC cycles. Dose reduction was not allowed, but temporary interruption was permitted for toxicity management. PD-1 inhibitors were permanently discontinued for radiographic progression, unacceptable toxicity, death, or patient withdrawal.

### 2.5 Patient assessment and follow-up

Follow-up included medical history, physical examination, laboratory tests, electrocardiography, chest CT, and contrast-enhanced abdominal CT or MRI. Tumor response was assessed every 6 weeks (after two HAIC cycles) and every 2 months during the systemic therapy maintenance phase. Best overall response (BOR) was independently evaluated by two experienced abdominal radiologists according to RECIST version 1.1, with discrepancies resolved by consensus. Adverse events were graded using Common Terminology Criteria for Adverse Events (CTCAE) version 5.0.

Primary endpoints were objective response rate (ORR), progression-free survival (PFS), overall survival (OS), and safety; the secondary endpoint was disease control rate (DCR). ORR was defined as the proportion of patients achieving complete response (CR) or partial response (PR). DCR included CR, PR, or stable disease (SD). PFS was the time from treatment initiation to radiographic progression or death, whichever came first. OS was the time from treatment start to death from any cause. For non-measurable PVTT lesions, response was evaluated using operational criteria adapted from prior studies (18, 23, 24) because of the lack of standardized assessment methods. CR was defined as complete resolution of thrombus enhancement or complete portal vein recanalization; PR as partial resolution of enhancement or downstaging according to JSH VP classification; progressive disease (PD) as increased PVTT extent with persistent enhancement or upstaging according to JSH VP classification; all other cases were classified as SD. These criteria applied to PVTT assessment only. After progression, subsequent treatments were determined by a multidisciplinary tumor board (MDT).

### 2.6 Statistical analyses

Continuous variables were expressed as mean ± standard deviation (SD) or median (interquartile range [IQR]) and compared using the Student t-test or Mann–Whitney U test, as appropriate. Categorical data were presented as numbers (percentages) and analyzed using the chi-square test or Fisher exact test. To reduce selection bias, stabilized inverse probability of treatment weighting (sIPTW) was applied based on propensity scores derived from a multivariable logistic regression model. Covariate balance was assessed using standardized mean differences (SMD); an absolute SMD < 0.1 was considered to indicate adequate balance. Survival outcomes were estimated using the Kaplan–Meier method and compared using the weighted log-rank test. Weighted Cox proportional hazards models were used to estimate hazard ratios (HRs) with 95% confidence intervals (CIs). Because the two treatment strategies had partially non-overlapping enrollment periods, a sensitivity analysis restricted to the shared enrollment window (June 2019 to September 2021) was performed to evaluate potential temporal bias. In this subset, PFS and OS were reanalyzed using Kaplan–Meier estimation and Cox proportional hazards models. All analyses were performed using R software version 4.5.2 (The R Foundation for Statistical Computing). A two-sided P value < 0.05 was considered statistically significant.

## 3 Results

### 3.1 Patient enrollment and baseline characteristics

**Figure 1:**
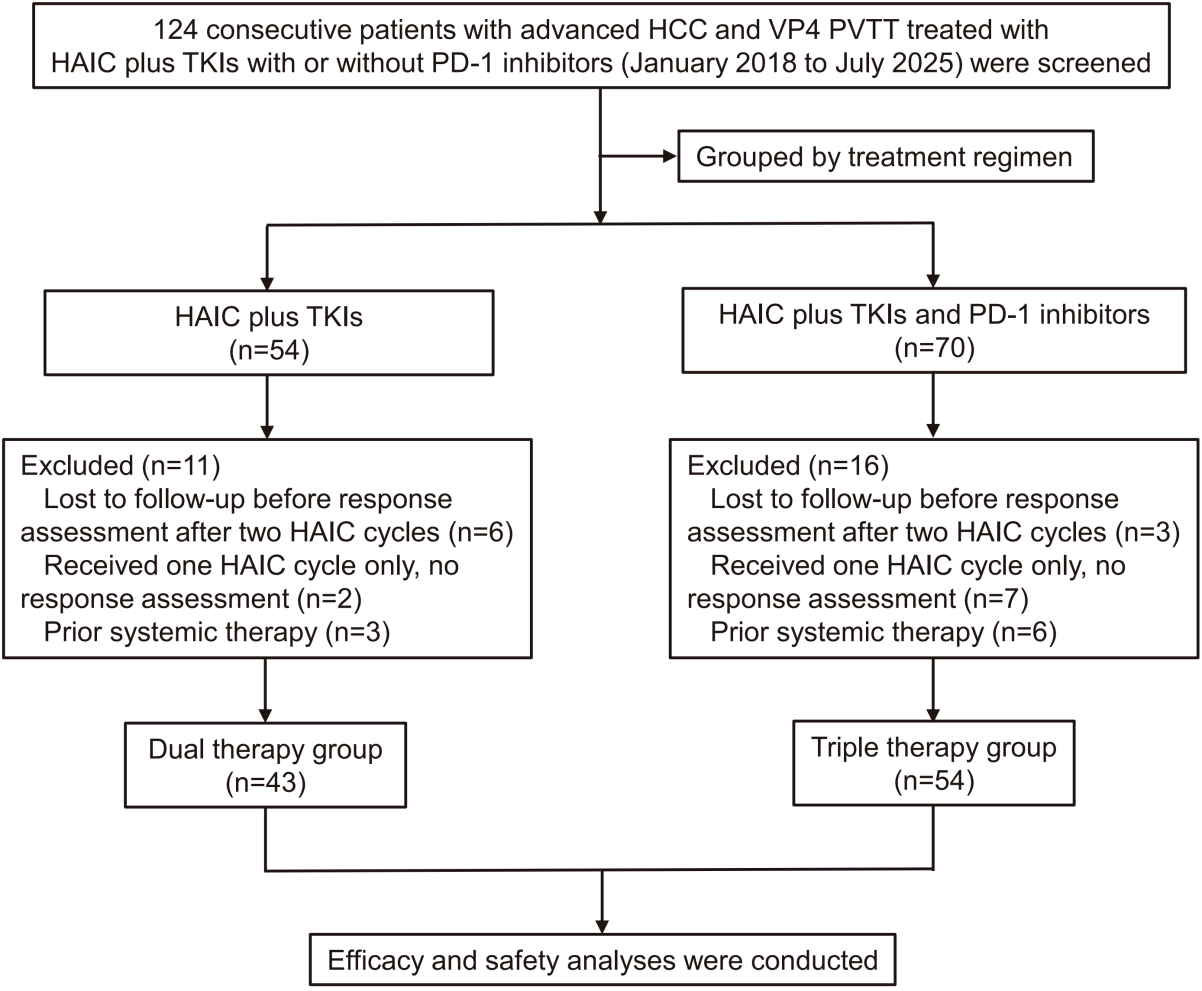
Patient selection and enrollment flowchart. A total of 124 consecutive patients with advanced HCC and VP4 PVTT were screened, and 97 eligible treatment-naïve patients were finally included in the analysis, with 43 in the dual therapy group and 54 in the triple therapy group. HCC, hepatocellular carcinoma; PVTT, portal vein tumor thrombus; VP4, Vp4 according to the Japanese classification; HAIC, hepatic arterial infusion chemotherapy; TKI, tyrosine kinase inhibitor; PD-1, programmed cell death protein 1.

**Figure 1** illustrates the patient enrollment process. A total of 124 consecutive patients with advanced HCC and VP4 PVTT treated with HAIC plus TKIs, with or without PD-1 inhibitors, were screened. After excluding 27 patients, 97 were included in the final analysis: 43 in the dual therapy group (HAIC plus TKIs) and 54 in the triple therapy group (HAIC plus TKIs and PD-1 inhibitors). The data cutoff date was December 31, 2025. Median follow-up for the entire cohort was 42.3 months (95% CI 37.3–60.0), as calculated by the reverse Kaplan–Meier method. Median follow-up was longer in the dual therapy group (68.7 months; 95% CI not estimable [NE]) than in the triple therapy group (41.7 months; 95% CI 34.1–50.7; P = 0.005). This difference reflects the earlier enrollment period of the dual therapy cohort. Nevertheless, median follow-up exceeding 3 years in the triple therapy group was sufficient for survival analysis.

Baseline clinical characteristics of the two groups before and after sIPTW are summarized in **Table 1**. No PD-1 inhibitors were administered in the dual therapy group. In the triple therapy group, PD-1 inhibitors included camrelizumab (n=23, 42.6%), tislelizumab (n=20, 37.0%), sintilimab (n=10, 18.5%), and pembrolizumab (n=1, 1.9%).

**Table 1:** Baseline characteristics before and after sIPTW adjustment.

Characteristic	Before sIPTW				After sIPTW			
	Dual therapy (n = 43)	Triple therapy (n = 54)	*P* Value	SMD	Dual therapy (n = 43.8)	Triple therapy (n = 53.0)	*P* Value	SMD
Age, years, mean (SD)	50.26 (9.89)	53.37 (9.91)	0.127	0.315	51.90 (9.65)	52.22 (10.09)	0.878	0.033
Sex, n (%)			0.295	0.277			0.867	0.034
Female	7 (16.3)	4 (7.4)			4.4 (10.1)	4.8 (9.1)		
Male	36 (83.7)	50 (92.6)			39.4 (89.9)	48.2 (90.9)		
ECOG PS, n (%)			1.000	0.069			0.957	0.010
0–1	39 (90.7)	50 (92.6)			40.9 (93.3)	49.3 (93.1)		
2	4 (9.3)	4 (7.4)			2.9 (6.7)	3.7 (6.9)		
Etiology, n (%)			0.841	0.158			0.776	0.052
Other	2 (4.7)	1 (1.9)			1.2 (2.6)	1.0 (1.9)		
HBV	41 (95.3)	53 (98.1)			42.6 (97.4)	52.0 (98.1)		
Child-Pugh class, n (%)			0.282	0.264			0.967	0.009
A	24 (55.8)	37 (68.5)			28.6 (65.4)	34.9 (65.8)		
B	19 (44.2)	17 (31.5)			15.2 (34.6)	18.1 (34.2)		
ALBI grade, n (%)			0.578	0.171			0.925	0.022
1	6 (14.0)	11 (20.4)			8.8 (20.1)	10.2 (19.2)		
2	37 (86.0)	43 (79.6)			35.0 (79.9)	42.8 (80.8)		
NLR, median (IQR)	3.17 (2.12–4.16)	2.83 (2.02–4.06)	0.736	0.131	3.18 (2.20–4.03)	2.67 (1.98–3.64)	0.284	0.038
PLR, median (IQR)	145.00 (101.38–171.00)	137.62 (97.84–173.76)	0.965	0.045	135.57 (89.20–172.58)	131.58 (95.28–170.44)	0.853	0.010
AFP, ng/mL, median (IQR)	5134.00 (287.70–81553.00)	1165.50 (48.17–46355.00)	0.281	0.046	4087.06 (95.98–47883.39)	976.84 (30.98–41271.69)	0.305	0.023
HAPF, n (%)			0.490	0.195			0.976	0.007
Absent	36 (83.7)	41 (75.9)			34.3 (78.3)	41.6 (78.5)		
Present	7 (16.3)	13 (24.1)			9.5 (21.7)	11.4 (21.5)		
Tumor size, cm, mean (SD)	10.96 (3.32)	10.58 (4.26)	0.635	0.099	10.69 (3.32)	10.71 (4.34)	0.980	0.005
Tumor number, n (%)			0.884	0.079			0.907	0.025
≤3	11 (25.6)	12 (22.2)			10.7 (24.3)	13.5 (25.4)		
>3	32 (74.4)	42 (77.8)			33.1 (75.7)	39.5 (74.6)		
EHS, n (%)			0.855	0.081			0.781	0.061
No	27 (62.8)	36 (66.7)			27.0 (61.6)	34.2 (64.6)		
Yes	16 (37.2)	18 (33.3)			16.8 (38.4)	18.8 (35.4)		
TKI type, n (%)			0.256	0.277			0.917	0.023
Lenvatinib	18 (41.9)	30 (55.6)			23.4 (53.4)	27.7 (52.3)		
Apatinib	25 (58.1)	24 (44.4)			20.4 (46.6)	25.3 (47.7)		
HAIC cycles, median (IQR)	4.00 (2.00–4.00)	4.00 (3.00–4.00)	0.515	0.104	4.00 (2.00–4.00)	4.00 (2.95–4.00)	0.599	0.094
PD-1 inhibitor type, n (%)			—	—			—	—
Camrelizumab	—	23 (42.6)			—	—		
Tislelizumab	—	20 (37.0)			—	—		
Sintilimab	—	10 (18.5)			—	—		
Pembrolizumab	—	1 (1.9)			—	—		
Later-line therapy, n (%)			0.965	0.051			0.874	0.034
No	21 (48.8)	25 (46.3)			19.1 (43.5)	23.9 (45.2)		
Yes	22 (51.2)	29 (53.7)			24.7 (56.5)	29.1 (54.8)		

Data are presented as number (%), mean (SD), or median (IQR). P values were calculated using the Student t-test, Mann-Whitney U test, chi-square test, or Fisher exact test as appropriate. SMD, standardized mean difference; sIPTW, stabilized inverse probability of treatment weighting; ECOG PS, Eastern Cooperative Oncology Group performance status; HBV, hepatitis B virus; ALBI, albumin-bilirubin; NLR, neutrophil-to-lymphocyte ratio; PLR, platelet-to-lymphocyte ratio; AFP, α-fetoprotein; HAPF, hepatic arterioportal fistula; EHS, extrahepatic spread; TKI, tyrosine kinase inhibitor; HAIC, hepatic arterial infusion chemotherapy; PD-1, programmed cell death protein 1; IQR, interquartile range.

Covariates included in the sIPTW model were age, sex, ECOG PS, etiology, Child-Pugh class, albumin-bilirubin (ALBI) grade, neutrophil-to-lymphocyte ratio (NLR), platelet-to-lymphocyte ratio (PLR), α-fetoprotein (AFP) level, presence of hepatic arterioportal fistula (HAPF), tumor size, tumor number, extrahepatic spread (EHS), and TKI type. Before weighting, several key covariates had moderate imbalances (SMD>0.2): patients in the triple therapy group were more likely to be male (92.6% vs 83.7%; SMD = 0.277), slightly older (mean age 53.37 vs 50.26 years; SMD = 0.315), had better liver function (Child-Pugh class A: 68.5% vs 55.8%; SMD = 0.264), and more frequently received lenvatinib (55.6% vs 41.9%; SMD = 0.277). Although these differences did not reach statistical significance due to limited sample size, the SMDs suggested potential confounding. Variables not included in the sIPTW model (number of HAIC cycles and distribution of later-line therapy) showed no significant between-group differences (P = 0.515 and P = 0.965, respectively).

**Figure 2:**
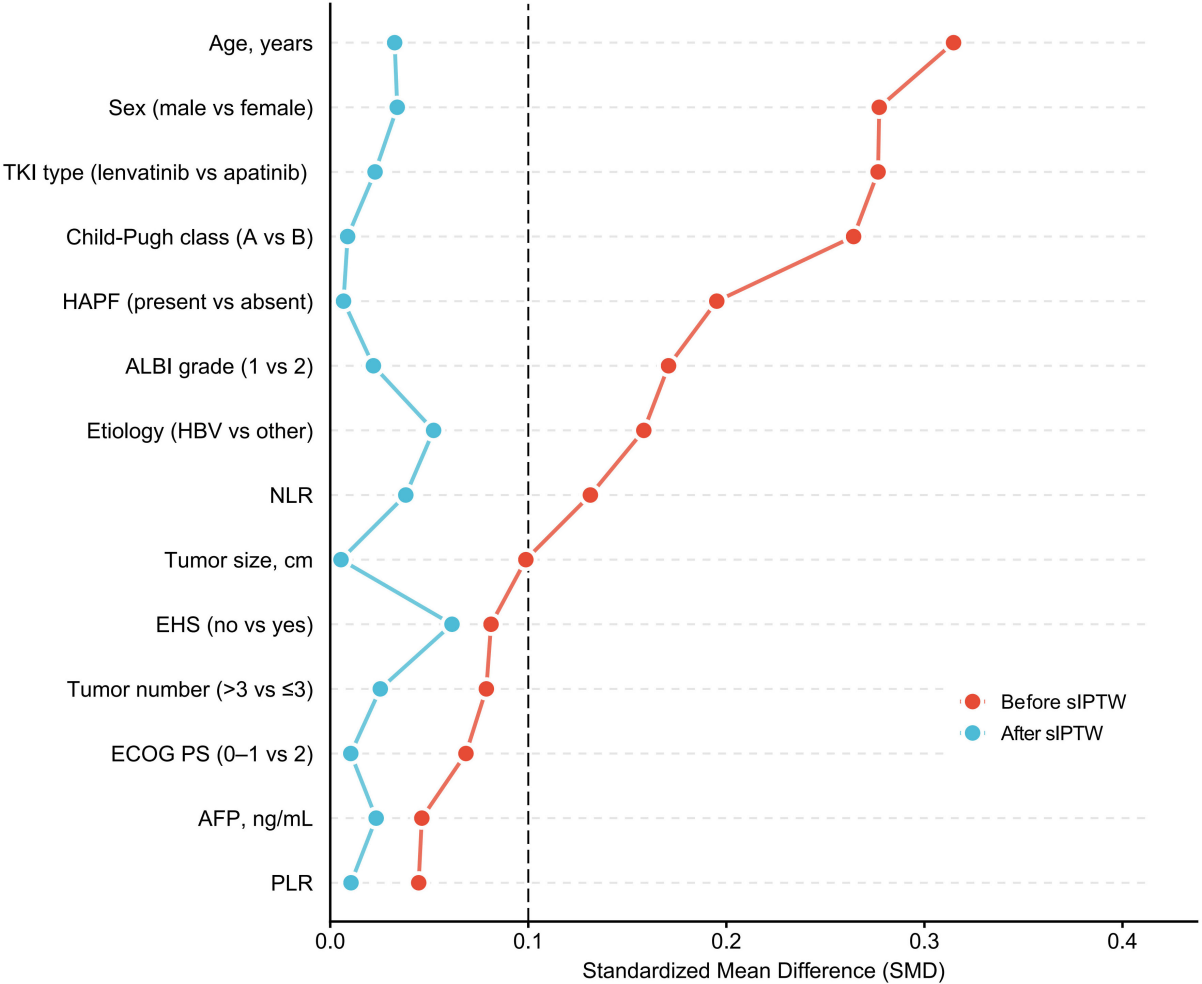
Love plot of baseline covariate balance before and after sIPTW adjustment. The love plot demonstrates the standardized mean difference (SMD) for baseline covariates before (red) and after (blue) sIPTW adjustment. The vertical dashed line at SMD = 0.10 indicates the threshold for adequate balance. After sIPTW adjustment, all covariates achieved SMD values below 0.10, indicating successful balance between the dual therapy and triple therapy groups. sIPTW, stabilized inverse probability of treatment weighting; TKI, tyrosine kinase inhibitor; HAPF, hepatic arterioportal fistula; ALBI, albumin-bilirubin; HBV, hepatitis B virus; NLR, neutrophil-to-lymphocyte ratio; EHS, extrahepatic spread; ECOG PS, Eastern Cooperative Oncology Group performance status; AFP, α-fetoprotein; PLR, platelet-to-lymphocyte ratio; SMD, standardized mean difference.

After sIPTW adjustment, all covariates were well balanced between the two groups, with all SMD values <0.1 and all between-group P values >0.05. Covariate balance before and after weighting is shown in the Love plot (**Figure 2**).

### 3.2 Tumor and PVTT response assessment

Tumor response was evaluated according to RECIST version 1.1, and PVTT response was assessed simultaneously, with results presented in **Table 2**. Before sIPTW adjustment, the triple therapy group had a higher ORR than the dual therapy group (61.2% vs 32.6%, P = 0.010) and a higher DCR (94.5% vs 67.4%, P = 0.001). sIPTW-adjusted analysis confirmed these findings, with an ORR of 58.9% vs 31.4% (P = 0.012) and a DCR of 94.8% vs 72.1% (P = 0.003) for triple therapy. For PVTT response, the triple therapy group also had a higher ORR than the dual therapy group before sIPTW (51.9% vs 25.6%, P = 0.016), with a PVTT CR rate of 13.0% vs 4.7%. This advantage persisted after sIPTW adjustment (ORR 49.9% vs 26.5%, P = 0.034). PVTT DCR was numerically higher in the triple therapy group both before (96.3% vs 88.4%, P = 0.270) and after sIPTW (96.6% vs 88.1%, P = 0.111), although the difference was not statistically significant.

**Table 2:** Tumor and PVTT response assessment.

Response	Before sIPTW			After sIPTW		
	Dual therapy (n = 43)	Triple therapy (n = 54)	*P* value	Dual therapy (n = 43.8)	Triple therapy (n = 53.0)	*P* value
Tumor response (RECIST version 1.1)						
CR	1 (2.3)	3 (5.6)				
PR	13 (30.2)	30 (55.6)				
SD	15 (34.9)	18 (33.3)				
PD	14 (32.6)	3 (5.6)				
ORR	14 (32.6)	33 (61.2)	0.010	13.7 (31.4)	31.2 (58.9)	0.012
DCR	29 (67.4)	51 (94.5)	0.001	31.6 (72.1)	50.2 (94.8)	0.003
PVTT Response						
CR	2 (4.7)	7 (13.0)				
PR	9 (20.9)	21 (38.9)				
SD	27 (62.8)	24 (44.4)				
PD	5 (11.6)	2 (3.7)				
ORR	11 (25.6)	28 (51.9)	0.016	11.6 (26.5)	26.5 (49.9)	0.034
DCR	38 (88.4)	52 (96.3)	0.270	38.5 (88.1)	51.2 (96.6)	0.111

Data are presented as number (%). Tumor response was assessed according to RECIST version 1.1. PVTT response was evaluated simultaneously. P values were calculated using the chi-square test or Fisher exact test as appropriate. RECIST, Response Evaluation Criteria in Solid Tumors; PVTT, portal vein tumor thrombus; sIPTW, stabilized inverse probability of treatment weighting; CR, complete response; PR, partial response; SD, stable disease; PD, progressive disease; ORR, objective response rate; DCR, disease control rate.

### 3.3 Survival outcomes

**Figure 3:**
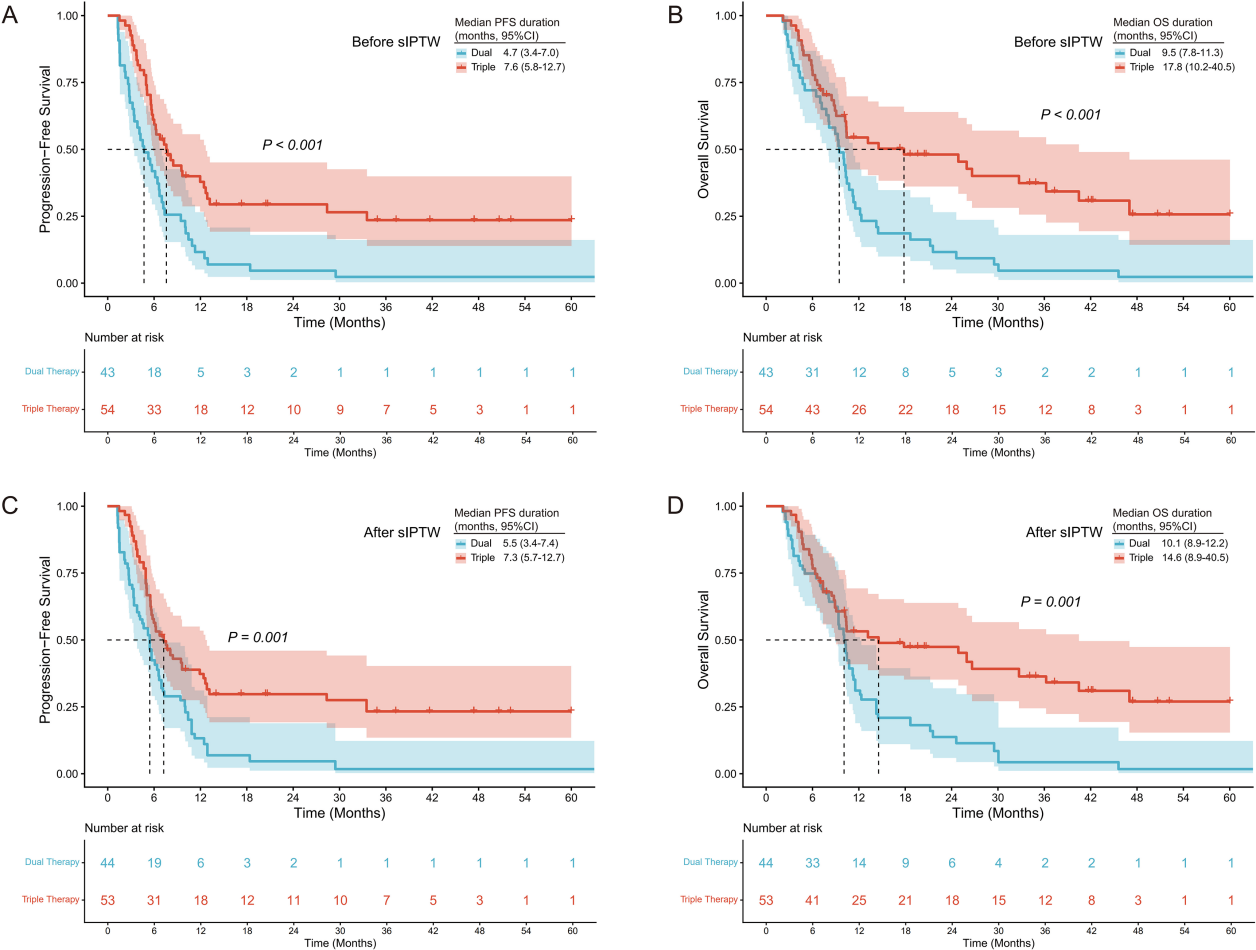
Kaplan-Meier survival curves for PFS and OS before **(A, B)** and after **(C, D)** sIPTW adjustment. Kaplan-Meier curves showing PFS and OS in the dual therapy and triple therapy groups. Before sIPTW adjustment **(A, B)**, triple therapy showed significantly longer median PFS (7.6 vs 4.7 months, P<0.001) and OS (17.8 vs 9.5 months, P<0.001). After sIPTW adjustment **(C, D)**, the survival benefit persisted with median PFS of 7.3 versus 5.5 months (P = 0.001) and median OS of 14.6 versus 10.1 months (P = 0.001). PFS, progression-free survival; OS, overall survival; sIPTW, stabilized inverse probability of treatment weighting; CI, confidence interval.

Kaplan–Meier curves for PFS and OS before and after sIPTW adjustment are shown in **Figure 3**. In the unweighted cohort, median PFS was 4.7 months (95% CI 3.4–7.0) for dual therapy and 7.6 months (95% CI 5.8–12.7) for triple therapy (HR 0.45, 95% CI 0.29–0.70, P<0.001). Median OS was 9.5 months (95% CI 7.8–11.3) for dual therapy and 17.8 months (95% CI 10.2–40.5) for triple therapy (HR 0.44, 95% CI 0.27–0.69, P<0.001). After sIPTW adjustment, median PFS was 5.5 months (95% CI 3.4–7.4) for dual therapy and 7.3 months (95% CI 5.7–12.7) for triple therapy (HR 0.48, 95% CI 0.31–0.75, P = 0.001). Median OS was 10.1 months (95% CI 8.9–12.2) for dual therapy and 14.6 months (95% CI 8.9–40.5) for triple therapy (HR 0.47, 95% CI 0.29–0.74, P = 0.001).

To evaluate potential temporal bias, a sensitivity analysis was restricted to patients treated during the overlapping enrollment period (June 2019 to September 2021). In this subset, triple therapy showed a trend toward longer PFS than dual therapy, though the difference did not reach statistical significance (HR 0.70, 95% CI 0.39–1.26, P = 0.235). The OS advantage with triple therapy remained statistically significant (HR 0.53, 95% CI 0.29–0.97, P = 0.039) (**Supplementary Figure 1**).

### 3.4 Subgroup analyses

**Figure 4:**
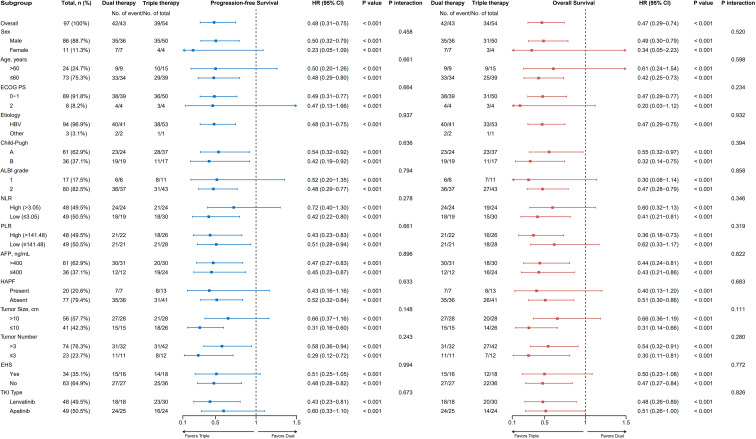
Forest plots of subgroup analyses for PFS and OS based on sIPTW-weighted cohort. Forest plots showing hazard ratios (HRs) with 95% confidence intervals (CIs) for the effect of triple therapy versus dual therapy on progression-free survival (PFS, left panel) and overall survival (OS, right panel) across predefined subgroups in the sIPTW-weighted cohort. The vertical dashed line at HR = 1.0 indicates no difference between treatment groups. HRs <1.0 favor triple therapy. All subgroups demonstrated consistent benefit from triple therapy for both PFS and OS (all P<0.001), with no significant treatment-by-subgroup interactions detected (all P for interaction >0.10). PFS, progression-free survival; OS, overall survival; sIPTW, stabilized inverse probability of treatment weighting; HR, hazard ratio; CI, confidence interval; ECOG PS, Eastern Cooperative Oncology Group performance status; HBV, hepatitis B virus; ALBI, albumin-bilirubin; NLR, neutrophil-to-lymphocyte ratio; PLR, platelet-to-lymphocyte ratio; AFP, α-fetoprotein; HAPF, hepatic arterioportal fistula; EHS, extrahepatic spread; TKI, tyrosine kinase inhibitor.

Subgroup analyses of PFS and OS based on the sIPTW-weighted cohort are presented in **Figure 4**. The benefit of triple therapy was consistent across nearly all predefined subgroups, with no significant treatment-by-covariate interaction observed (all P for interaction > 0.05). When stratified by TKI type, the treatment effect of triple therapy remained directionally consistent. For PFS, the HR was 0.43 (95% CI 0.23–0.81) in patients receiving lenvatinib and 0.60 (95% CI 0.33–1.10) in those receiving apatinib (P for interaction = 0.673). For OS, the corresponding HRs were 0.48 (95% CI 0.26–0.89) and 0.51 (95% CI 0.26–1.00), respectively (P for interaction = 0.826).

### 3.5 Prognostic factor analysis

Univariable and multivariable Cox regression analyses based on the sIPTW-weighted cohort identified independent prognostic factors for PFS and OS (**Table 3**). Triple therapy was an independent protective factor for both PFS (multivariable HR 0.45, 95% CI 0.29–0.70, P<0.001) and OS (multivariable HR 0.43, 95% CI 0.27–0.69, P<0.001). Elevated NLR was an independent adverse prognostic factor for PFS (multivariable HR 1.13, 95% CI 1.03–1.24, P = 0.013) and OS (multivariable HR 1.18, 95% CI 1.04–1.34, P = 0.008). Other baseline covariates, including sex, age, ECOG PS, Child-Pugh class, AFP level, and EHS, were not significantly associated with survival outcomes.

**Table 3:** Univariable and multivariable Cox regression analysis of prognostic factors for PFS and OS after sIPTW adjustment.

Characteristic	PFS				OS			
	Univariable analysis		Multivariable analysis		Univariable analysis		Multivariable analysis	
	HR (95% CI)	*P* Value	HR (95% CI)	*P V*alue	HR (95% CI)	*P* Value	HR (95% CI)	*P* Value
Treatment Regimen (triple vs dual)	0.48 (0.31–0.75)	0.001	0.45 (0.29–0.70)	<0.001	0.47 (0.29–0.74)	0.001	0.43 (0.27–0.69)	<0.001
Sex (Male vs Female)	0.71 (0.35–0.45)	0.345	—	—	0.85 (0.35–2.07)	0.715	—	—
Age, years (continuous)	0.99 (0.97–1.01)	0.431	—	—	0.99 (0.97–1.02)	0.508	—	—
ECOG PS (2 vs 0-1)	1.38 (0.51–3.72)	0.520	—	—	1.69 (0.63–4.48)	0.302	—	—
Etiology (HBV vs Other)	0.53 (0.25–1.16)	0.113	—	—	0.72 (0.43–1.22)	0.222	—	—
Child-Pugh class (B vs A)	0.94 (0.59–1.51)	0.802	—	—	1.20 (0.75–1.92)	0.453	—	—
ALBI grade (2 vs 1)	1.29 (0.78–2.13)	0.318	—	—	1.35 (0.82–2.23)	0.233	—	—
NLR (continuous)	1.09 (0.99–1.19)	0.072	1.13 (1.03–1.24)	0.013	1.13 (1.01–1.27)	0.035	1.18 (1.04–1.34)	0.008
PLR (continuous)	1.00 (1.00–1.00)	0.333	—	—	1.00 (1.00–1.01)	0.196	—	—
AFP, ng/ml (>400 vs ≤400)	0.94 (0.60–1.46)	0.781	—	—	0.87 (0.54–1.42)	0.582	—	—
HAPF (present vs absent)	0.88 (0.53–1.46)	0.624	—	—	1.14 (0.69–1.89)	0.615	—	—
Tumor size, cm (>10 vs ≤10)	1.19 (0.76–1.86)	0.442	—	—	1.33 (0.83–2.12)	0.236	—	—
Tumor number (>3 vs ≤3)	1.13 (0.65–1.97)	0.661	—	—	1.13 (0.61–2.11)	0.691	—	—
EHS (yes vs no)	1.00 (0.62–1.62)	0.986	—	—	0.95 (0.57–1.59)	0.847	—	—
TKI type (apatinib vs lenvatinib)	1.00 (0.64–1.56)	0.984	—	—	1.13 (0.71–1.81)	0.596	—	—

Hazard ratios and 95% confidence intervals were calculated using Cox proportional hazards regression. Variables with P<0.10 in univariable analysis were entered into the multivariable model. The first category listed is the comparison group, and the second is the reference. PFS, progression-free survival; OS, overall survival; sIPTW, stabilized inverse probability of treatment weighting; HR, hazard ratio; CI, confidence interval; ECOG PS, Eastern Cooperative Oncology Group performance status; HBV, hepatitis B virus; ALBI, albumin-bilirubin; NLR, neutrophil-to-lymphocyte ratio; PLR, platelet-to-lymphocyte ratio; AFP, α-fetoprotein; HAPF, hepatic arterioportal fistula; EHS, extrahepatic spread; TKI, tyrosine kinase inhibitor.

### 3.6 Safety

Treatment-related adverse events (TRAEs) were assessed in all enrolled patients and are summarized in **Table 4**. Any-grade TRAEs occurred in all patients in both groups (100.0%). Grade 3–4 TRAEs occurred in 13 patients (30.2%) in the dual therapy group and 19 patients (35.2%) in the triple therapy group, with no significant difference (P = 0.606). Hepatobiliary and hematologic events were the most common TRAEs in both groups. In the dual therapy group, the most frequent any-grade TRAEs were elevated AST (88.4%), elevated ALT (76.7%), thrombocytopenia (55.8%), and hyperbilirubinemia (51.2%). In the triple therapy group, the most frequent any-grade TRAEs were elevated AST (75.9%), thrombocytopenia (66.7%), elevated ALT (64.8%), and leukopenia (61.1%). These toxicities did not differ significantly between groups (all P>0.05). For grade 3–4 TRAEs, elevated AST (14.0%) and thrombocytopenia (11.6%) were most common in the dual therapy group, whereas thrombocytopenia (18.5%) and leukopenia (9.3%) were most common in the triple therapy group. Other TRAEs, including gastrointestinal symptoms, fatigue, hypertension, and fever, were generally grade 1–2 and comparable between groups. Immune-related adverse events (irAEs), including hypothyroidism, rash, hepatitis, and pneumonitis, occurred only in the triple therapy group; most were grade 1–2, except for one case each of grade 3 rash and grade 3 hepatitis. No treatment-related deaths occurred.

**Table 4:** Treatment-related adverse events.

Adverse event	Dual therapy (n=43)		Triple therapy (n=54)		*P* value	
	Any Grade	Grade 3–4	Any Grade	Grade 3–4	Any Grade	Grade 3–4
Overall incidence	43 (100.0)	13 (30.2)	54 (100.0)	19 (35.2)	—	0.606
Leukopenia	18 (41.9)	2 (4.7)	33 (61.1)	5 (9.3)	0.059	0.634
Thrombocytopenia	24 (55.8)	5 (11.6)	36 (66.7)	10 (18.5)	0.274	0.351
Elevated ALT	33 (76.7)	0 (0.0)	35 (64.8)	2 (3.7)	0.202	0.501^a^
Elevated AST	38 (88.4)	6 (14.0)	41 (75.9)	3 (5.6)	0.117	0.287
Hyperbilirubinemia	22 (51.2)	2 (4.7)	23 (42.6)	0 (0.0)	0.400	0.194^a^
Fever	4 (9.3)	0 (0.0)	12 (22.2)	0 (0.0)	0.089	—
Abdominal pain	12 (27.9)	1 (2.3)	17 (31.5)	1 (1.9)	0.702	1.000^a^
Nausea/vomiting	10 (23.3)	0 (0.0)	11 (20.4)	0 (0.0)	0.732	—
Diarrhea	7 (16.3)	1 (2.3)	9 (16.7)	0 (0.0)	0.959	0.443^a^
Gastric ulcer	0 (0.0)	0 (0.0)	2 (3.7)	2 (3.7)	0.501^a^	0.501^a^
Gastrointestinal bleeding	2 (4.7)	2 (4.7)	5 (9.26)	3 (5.6)	0.634	1.000
Fatigue	10 (23.3)	0 (0.00)	18 (33.3)	0 (0.0)	0.277	—
Hypertension	9 (20.9)	1 (2.3)	11 (20.4)	2 (3.7)	0.946	1.000
Oral ulcer	4 (9.3)	0 (0.0)	3 (5.6)	0 (0.0)	0.754	—
Hand-foot syndrome	5 (11.6)	0 (0.0)	3 (5.6)	0 (0.0)	0.479	—
Weight loss	9 (20.9)	0 (0.0)	13 (24.1)	0 (0.0)	0.713	—
Immune-related AEs						
Hypothyroidism	—	—	5 (9.3)	0 (0.0)	—	—
Rash	—	—	6 (11.1)	1 (1.9)	—	—
Hepatitis	—	—	2 (3.7)	1 (1.9)	—	—
Pneumonitis	—	—	2 (3.7)	0 (0.0)	—	—

Data are presented as number (%). Adverse events were graded according to CTCAE version 5.0. P values were calculated using the chi-square test or Fisher exact test as appropriate. ^a^Fisher exact test. HAIC, hepatic arterial infusion chemotherapy; TKI, tyrosine kinase inhibitor; ALT, alanine aminotransferase; AST, aspartate aminotransferase; CTCAE, Common Terminology Criteria for Adverse Events.

## 4 Discussion

HCC with VP4 PVTT has a poor prognosis and responds poorly to systemic therapy alone, representing one of the most challenging scenarios in hepatobiliary oncology. Combining HAIC with systemic agents has attracted growing interest for this high-risk population. To our knowledge, this is the first study to directly compare triple therapy (HAIC plus TKIs and PD-1 inhibitors) with dual therapy (HAIC plus TKIs) in patients with VP4 PVTT. Our results showed that triple therapy achieved a higher ORR and improved PFS and OS, without increased severe toxicity. These findings suggest that adding PD-1 inhibitors to HAIC plus TKIs may improve outcomes and refine treatment strategies for patients with VP4 PVTT.

In this study, the triple therapy group achieved a sIPTW-adjusted median OS of 14.6 months and median PFS of 7.3 months. These outcomes are notable in the VP4 PVTT setting and consistent with recent reports of similar triple-therapy regimens. In a phase II trial of HAIC plus lenvatinib and toripalimab enrolling patients with high-risk advanced HCC including VP4 PVTT, Lai et al. (19) reported a median OS of 17.9 months and PFS of 10.4 months. Li et al. (25) observed a median OS of 16.7 months and PFS of 8.0 months in patients with VP4 PVTT receiving HAIC plus lenvatinib and tislelizumab, while Tang et al. (26), using the same regimen in a similar cohort, reported a median OS of 23.2 months and PFS of 6.6 months. Li et al. (27) also reported a median OS of 21.2 months and PFS of 7.4 months with HAIC plus lenvatinib and toripalimab in patients with large (>10 cm) HCC and major PVTT. Together, these results support the clinical potential of triple therapy in this refractory population. The variation in survival estimates across studies likely reflects differences in baseline characteristics, including liver function reserve, intrahepatic tumor burden, and the presence of extrahepatic metastasis, as well as the specific systemic agents used. Compared with dual therapy, the survival advantage of triple therapy in our cohort was substantial. The sIPTW-adjusted median OS in the dual therapy group was 10.1 months, shorter than the 13.4–16.3 months reported for HAIC plus sorafenib in broader PVTT populations (16, 17). This difference likely reflects our strict restriction to VP4 PVTT, which has a worse prognosis than lower-grade PVTT. Nevertheless, the 14.6-month median OS with triple therapy represents a meaningful improvement and compares favorably with historical outcomes of standard systemic therapy in this high-risk setting. To account for potential confounding from different enrollment periods between groups, we performed a sensitivity analysis restricted to the overlapping treatment window. In this analysis, the PFS benefit was attenuated and no longer statistically significant, likely owing to the reduced sample size and limited statistical power. The OS advantage of triple therapy, however, remained significant, suggesting that the observed survival benefit is attributable to the addition of PD-1 inhibitors rather than to temporal changes in supportive care or other period effects.

The Kaplan–Meier OS curves, both before and after sIPTW adjustment (**Figures 3B, D**), showed limited separation during early follow-up, with the difference becoming apparent only after approximately 12 months. Beyond that point, the triple therapy group demonstrated relative plateauing, whereas the dual therapy group continued to decline steeply. This pattern of late separation is commonly observed with immunotherapy and distinguishes it from conventional chemotherapy and targeted therapy (28). Mechanistically, immune checkpoint blockade can activate endogenous antitumor immunity and promote durable immune surveillance through memory T-cell responses (29, 30). Although HAIC plus TKIs can achieve rapid intrahepatic tumor control, their long-term benefit may be limited by the immunosuppressive tumor microenvironment (TME) characteristic of HCC. PD-1 inhibitors may overcome this limitation, as preclinical evidence indicates that oxaliplatin can induce immunogenic cell death (ICD), while VEGFR/FGFR inhibition (e.g., lenvatinib) may remodel the TME and enhance cytotoxic T-cell infiltration, thereby sensitizing tumors to PD-1 blockade (31, 32). These complementary mechanisms explain the more durable survival observed with triple therapy.

For therapeutic response, the sIPTW-adjusted ORR assessed by RECIST version 1.1 was 58.9% in the triple therapy group, higher than the 31.4% in the dual therapy group (P = 0.012). Of note, RECIST version 1.1 was selected for response assessment to maintain consistency with pivotal trials of systemic therapy for advanced HCC and to provide a standardized evaluation applicable to both intrahepatic and extrahepatic lesions in this retrospective cohort. This ORR is comparable to the 52%–77% reported in recent studies of HAIC combined with TKIs and PD-1 inhibitors for advanced HCC with PVTT (18–20, 25–27), despite our strict enrollment of only patients with the extremely high-risk VP4 PVTT subtype. Although cross-trial comparisons should be interpreted with caution, the ORR with triple therapy appears higher than that reported for contemporary first-line systemic regimens (approximately 23%–36% by RECIST version 1.1) (8–11, 33) and for HAIC plus TKI dual therapy in PVTT populations (e.g., approximately 41% in randomized trials of HAIC plus sorafenib and 45.7% in the LEOPARD study of HAIC plus lenvatinib) (16, 17, 34), suggesting potent tumor-shrinkage activity. Furthermore, the triple therapy group achieved a higher sIPTW-adjusted PVTT ORR (49.9% vs 26.5%, P = 0.034) and a numerically higher PVTT CR rate (13.0% vs 4.7%) than the dual therapy group. Achieving an effective PVTT response is clinically important, as it can restore portal venous flow, alleviate portal hypertension-related complications, and potentially enable tumor downstaging to curative-intent resection or ablation in selected patients.

Multivariable Cox regression analysis based on the sIPTW-weighted cohort identified treatment regimen and baseline NLR as independent prognostic factors for both PFS and OS. Triple therapy was independently associated with improved PFS (HR 0.45, 95% CI 0.29–0.70, P<0.001) and OS (HR 0.43, 95% CI 0.27–0.69, P<0.001), suggesting that the benefit of PD-1 inhibitor addition is not fully explained by imbalances in measured covariates, although residual confounding cannot be excluded. NLR, a readily available biomarker reflecting systemic inflammation and immune status, has established prognostic value across solid tumors (35, 36), and our findings are consistent with prior reports supporting its use for risk stratification in HAIC-based treatment settings (37, 38). Because treatment-by-NLR interactions were not formally tested, these data should be interpreted as prognostic rather than predictive of differential benefit from PD-1 inhibitor addition. Subgroup analyses showed consistent treatment effects across prespecified strata, with no significant interactions for any baseline covariate. However, these exploratory findings should be interpreted cautiously given limited power in several subgroups. The observed benefit in patients with Child–Pugh class B liver function is hypothesis-generating and requires prospective validation. The treatment effect was also directionally consistent across TKI subgroups, with no significant interaction between treatment strategy and TKI type for either PFS or OS. In the lenvatinib-only subgroup, triple therapy retained its advantage over dual therapy for both PFS (HR 0.43) and OS (HR 0.48), further supporting that the survival benefit is driven by the addition of PD-1 inhibitors rather than by differences in TKI selection between groups.

Safety is a prerequisite for broader adoption of intensive combination regimens. In our cohort, the incidence of grade 3–4 TRAEs did not differ between the triple and dual therapy groups (35.2% vs 30.2%, P = 0.606), indicating no increased severe toxicity with PD-1 inhibitor addition. The predominant toxicities in the dual therapy group were consistent with those expected from HAIC (e.g., myelosuppression and hepatic function impairment) and TKIs (e.g., hypertension and hand-foot skin reaction), consistent with prior reports (16, 17, 34). In the triple therapy group, immune-related adverse events, including hypothyroidism, rash, immune-mediated hepatitis, and pneumonitis, were also observed; most were grade 1–2 and manageable with temporary treatment interruption and standard supportive measures, and no treatment-related deaths occurred. Overall, the safety profile is consistent with that reported in other HAIC plus TKI and PD-1 inhibitor studies (19, 20, 26, 27), supporting the feasibility of this approach in appropriately selected patients.

Several limitations should be acknowledged. First, the retrospective, nonrandomized design is inherently susceptible to selection bias and residual confounding despite sIPTW adjustment and multivariable analysis, particularly temporal bias arising from partially different enrollment periods between groups, which the overlapping-period sensitivity analysis mitigated but cannot entirely exclude. Second, the single-center setting may limit generalizability. Third, the sample size was modest for this uncommon population, and subgroup analyses should be interpreted with caution. Fourth, the use of different TKI types and dosages, different PD-1 inhibitors, and non-protocolized post-progression therapies may have introduced treatment heterogeneity. However, subgroup analysis confirmed that the survival advantage persisted within the lenvatinib-only subgroup, reducing concerns about TKI selection bias. Regarding PD-1 inhibitors, the agents used in this study have each been approved for advanced HCC based on phase III trial data and are broadly regarded as having a class effect. In routine practice, the choice among these agents is largely determined by drug availability, insurance coverage, and patient affordability rather than by mechanistic differences. Grouping them as a single category therefore reflects real-world practice, though residual heterogeneity cannot be ruled out. Fifth, the PVTT response criteria were adapted from prior studies rather than from a validated standard and require further external validation. Despite these limitations, this study provides the first direct comparison of HAIC plus TKIs with versus without PD-1 inhibitors in VP4 PVTT, offering preliminary evidence to inform treatment selection. Prospective multicenter studies with standardized regimens and contemporaneous controls are needed to confirm these findings.

## 5 Conclusion

In patients with advanced HCC and VP4 PVTT, the addition of PD-1 inhibitors to HAIC plus TKIs was associated with improved tumor response and prolonged survival, without an apparent increase in severe treatment-related adverse events. These findings support triple therapy as a potentially preferred first-line treatment strategy for this high-risk population. Prospective multicenter studies, ideally with randomized designs and standardized regimens, are needed to validate these findings and optimize patient selection.

## Data Availability

The datasets generated and analyzed in this study are not publicly available due to ethical and privacy restrictions but are available from the corresponding author upon reasonable request. Requests to access the datasets should be directed to Weifu Liu, 361892418@qq.com.

## Supplementary material

Supplementary Figure 1 Kaplan–Meier Survival Curves for PFS **(A)** and OS **(B)** in the Overlapping Enrollment Period Cohort Kaplan–Meier curves showing PFS and OS restricted to patients enrolled during the overlapping calendar period between the dual-therapy and triple-therapy groups. Triple therapy showed a trend toward longer median PFS, though not statistically significant (HR = 0.70, 95% CI 0.39–1.26, P = 0.235). The OS benefit remained significant (HR = 0.53, 95% CI 0.29–0.97, P = 0.039). PFS, progression-free survival; OS, overall survival; HR, hazard ratio; CI, confidence interval.
