# Supplementary material for: Hepatic arterial infusion chemotherapy plus tyrosine kinase inhibitors with or without PD-1 inhibitors for advanced hepatocellular carcinoma with VP4 portal vein tumor thrombosis: a retrospective cohort study
Source: Front Immunol. 2026 May 20;17:1832313. doi: 10.3389/fimmu.2026.1832313 (PMC13229895; doi:10.3389/fimmu.2026.1832313)
Supplement: Supplementary Figure 1 — Kaplan–Meier Survival Curves for PFS (A) and OS (B) in the Overlapping Enrollment Period Cohort Kaplan–Meier curves showing PFS and OS restricted to patients enrolled during the overlapping calendar period between the dual-therapy and triple-therapy groups. Triple therapy showed a trend toward longer median PFS, though not statistically significant (HR = 0.70, 95% CI 0.39–1.26, P = 0.235). The OS benefit remained significant (HR = 0.53, 95% CI 0.29–0.97, P = 0.039). PFS, progression-free survival; OS, overall survival; HR, hazard ratio; CI, confidence interval. [file Image1.pdf]

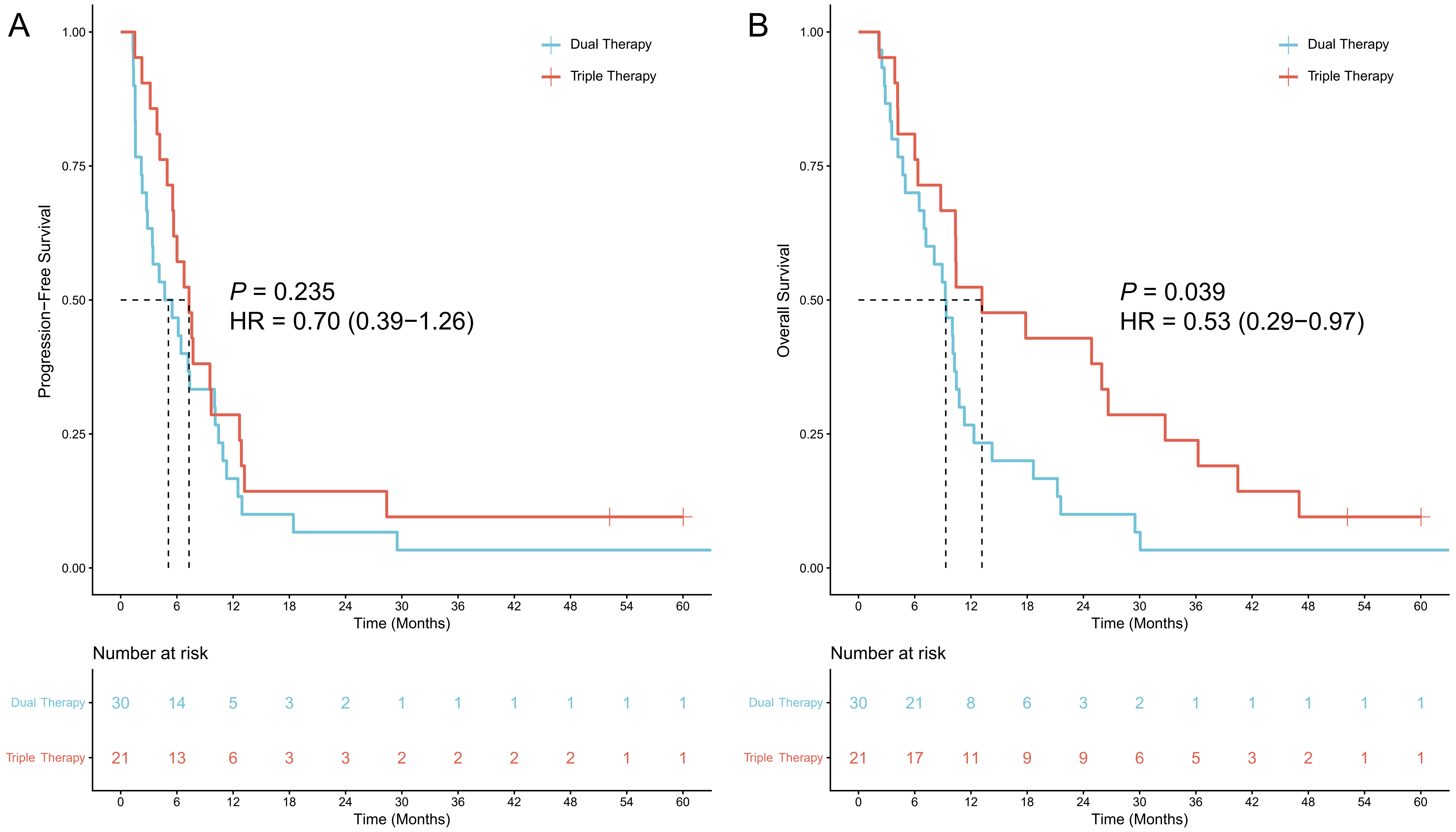

Supplementary Figure 1. Kaplan–Meier Survival Curves for PFS (A) and OS (B) in the Overlapping Enrollment Period Cohort.
Kaplan–Meier curves showing PFS and OS restricted to patients enrolled during the overlapping calendar period between the dual-therapy and triple-therapy groups. Triple therapy showed a trend toward longer median PFS, though not statistically significant (HR = 0.70, 95% CI 0.39–1.26, P = 0.235). The OS benefit remained significant (HR = 0.53, 95% CI 0.29–0.97, P = 0.039). PFS, progression-free survival; OS, overall survival; HR, hazard ratio; CI, confidence interval.
